# Accuracy of Response Time Data in EMS Systematic Review. Comment on Alruwaili, A.; Alanazy, A.R.M. Prehospital Time Interval for Urban and Rural Emergency Medical Services: A Systematic Literature Review. *Healthcare* 2022, *10*, 2391

**DOI:** 10.3390/healthcare14152292

**Published:** 2026-07-29

**Authors:** Fatemeh Mohammad Alizadeh Chafjiri

**Affiliations:** Division of Epilepsy and Clinical Neurophysiology, Department of Neurology Boston Children’s Hospital, Harvard Medical School, Boston, MA 02115, USA; fatemehmohammadalizadeh1993@gmail.com

I read your article, “Prehospital Time Interval for Urban and Rural Emergency Medical Services: A Systematic Literature Review” [1], with great interest. The topic is highly relevant, and your synthesis of the literature provides valuable insight into EMS response times across different settings.

While reviewing the tables, I noticed a possible point of confusion in Table 2 (Differences between rural and urban areas regarding response time). The table is described as presenting response times (defined in your study as the interval from receiving the alarm to arriving on-scene). However, the value of 3.7 min for urban areas attributed to the study by Lee et al. [2] seems to correspond to T1 (crash–reporting interval) in their work, rather than response time.

As I understand it, these intervals are distinct:

- Crash–reporting interval (T1): Time from crash occurrence → notification of EMS (e.g., calling 911).
- Response time: Time from EMS receiving the alarm/dispatch → EMS arrival on scene.

It seems that in the manuscript by Lee et al., response time was defined as T2 (reporting–scene interval, EMS notified → EMS arrives on-scene), which aligns with standard EMS terminology. Given this, the 3.7 min reported in Table 2 may not reflect response time as defined in your review, but rather T1 from the original study.

Could you please clarify whether this was the case? I believe this distinction would help readers interpret the data more accurately and avoid confusion regarding the intervals being compared across studies.

Thank you again for your valuable contribution to this field. I look forward to your response.

## Data Availability

No new data were created or analyzed in this study. Data sharing is not applicable to this article.
